# Heterogeneous graph construction and HinSAGE learning from electronic medical records

**DOI:** 10.1038/s41598-022-25693-2

**Published:** 2022-12-07

**Authors:** Ha Na Cho, Imjin Ahn, Hansle Gwon, Hee Jun Kang, Yunha Kim, Hyeram Seo, Heejung Choi, Minkyoung Kim, Jiye Han, Gaeun Kee, Tae Joon Jun, Young-Hak Kim

**Affiliations:** 1 Division of Cardiology, Department of Internal Medicine, Asan Medical Center, University of Ulsan College of Medicine, 88, Olympicro 43 Gil, Songpagu, 05505 Seoul, Republic of Korea; 2 Department of Medical Science, Asan Medical Institute of Convergence Science and Technology, Asan Medical Center, University of Ulsan College of Medicine, 88, Olympicro 43 Gil, Songpagu, 05505 Seoul, Republic of Korea; 3 Big Data Research Center, Asan Institute for Life Sciences, Asan Medical Center, 88, Olympicro 43 Gil, Songpagu, 05505 Seoul, Republic of Korea

**Keywords:** Medical research, Experimental models of disease

## Abstract

Graph representation learning is a method for introducing how to effectively construct and learn patient embeddings using electronic medical records. Adapting the integration will support and advance the previous methods to predict the prognosis of patients in network models. This study aims to address the challenge of implementing a complex and highly heterogeneous dataset, including the following: (1) demonstrating how to build a multi-attributed and multi-relational graph model (2) and applying a downstream disease prediction task of a patient’s prognosis using the *HinSAGE* algorithm. We present a bipartite graph schema and a graph database construction in detail. The first constructed graph database illustrates a query of a predictive network that provides analytical insights using a graph representation of a patient’s journey. Moreover, we demonstrate an alternative bipartite model where we apply the model to the *HinSAGE* to perform the link prediction task for predicting the event occurrence. Consequently, the performance evaluation indicated that our heterogeneous graph model was successfully predicted as a baseline model. Overall, our graph database successfully demonstrated efficient real-time query performance and showed *HinSAGE* implementation to predict cardiovascular disease event outcomes on supervised link prediction learning.

## Introduction

The management of healthcare data is constantly transforming to assist the healthcare industry in increasingly productive ways^1–3^. In traditional electronic medical record (EMR) systems, data are organized and managed in relational database systems, where there is no association between the data stored^4^. To illustrate, multiple tables are linked to each other with foreign keys attached in a column where the relation is not focused on the data points but between the data tables. Contrastingly, graph databases link the data records to organize data features more effectively by focusing more on the data points providing emphasis on the relationships^5,6^. In these, entities and links are used to increase space efficiency and provide a faster querying period for large datasets compared to relational mapping^7^. There are three major advantages to applying graph databases: Firstly, a graph allows the total visualization of the full picture, which delivers simplified or alternative perspectives to otherwise complex problems. Secondly, a graph enables a deeper understanding of abstract relationships. Lastly, a graph facilitates the understanding of the information flow and applies to improve the details.

A substantial number of studies have been performed on constructing graph representation learning in biomedicine^8–13^. However, EMR data currently include four characteristics that make it difficult to be converted into a network formation: (1) EMR has many different types of datasets, creating separate entity types. (2) Divergent datasets of node types have non-identical sizes and types of property sets. (3) Multiple types of edges are needed to correlate with the following entity types. (4) Multiple datasets are inherently linked by unique IDs. Due to the aforementioned reasons, applying the combined mechanism of multi-modal, multi-attributed, and multi-relational aspects of the EMR into the representation is still in development to fully convey the necessities of datasets into a graph structure.

Recently, considerable studies have been conducted in medicine focusing on building a knowledge graph or graph representations in medicine^14–18^. Indeed, a bipartite network database was constructed from electronic health records with heart failure patients^19^, and the network analysis of the relationships between patients and providers was demonstrated by calculating the network statistics. However, this study has shortcomings in spending a significant amount of time during the query. Moreover, a highly granular semantic knowledge graph built on rare diseases from EMR^20^ emphasized the importance of semi-automation schema creating more granular semantic relationships. The study produced increased concept correlations, nevertheless, the dataset includes less than a thousand tumor cases that lack validation. Alternatively, an embedding method of medical knowledge graphs using probability values was implemented for quadruplet structures^21^. Although this study proved that the prediction task performs better when the entity types are indicated, the evaluation dataset contained limited relationship types due to non-automatic labor-intensive works. Contrastingly, to supplement the effortful task, we created a graph-building process automated for labeling diverse link types alongside solved memory storage issues.

Moreover, to utilize the valuable assets from medical datasets, applications in a graph neural network are gaining popularity in personalized health and predictive medicine. For example, heterogeneous similarity graph neural network was used to analyze health records in terms of temporal structure aspects by forming multiple subgraphs as input for prediction^22^. Similarly, ME2Vec hierarchical graph predicts patients' clinical outcomes on the interactions of calculated entities^23^. However, to ease the graph learning process, we instead handled all hierarchical sequences during the pre-processing step and enabled its implementation regardless of data types and sizes. Moreover, In a graph model for population diagnosis, individual feature information was learned in binary classification tasks using graph convolutional networks^24^. To further extend the potential for applying attributes to the nodes, classification was performed at higher precision when distinct node attributes were added to each partition of a bipartite network^25–27^. Nonetheless, these researches used data source specific to claims data, which generally contain simple datasets compared to EMR data, thereby, limiting the data inclusion for reliable estimation in predictions. Additionally, studies have tackled integrating heterogeneous structured graphs combined with the attribute aspects^28–32^. Yet, the methods depicted in these studies do not fit well with the properties of the EMR in network integration. To elaborate, the EMR dataset has a unique structure that various types of datasets (medication, laboratory, physical, visits, etc.) are associated with each other to represent patients’ medical status and also connected by the anonymous key. However, previously mentioned graphs use genomics, proteomics, molecular biology, or movie review dataset that does not necessarily require the interconnection between the nodes, thereby, generating a network where attributes need no special linkage.

Here, we suggest the methodologies for the integration of heterogeneous medical entities and relationships to predict a patient’s outcome from a graph constructed based solely on the EMR dataset. Our main contributions are.

- The proposal of a novel approach to construct a heterogeneous bipartite graph model from EMR with attributes on nodes and edges. Using an effective visualization, in conjunction with a patient-centered graph method allows the latent associations among the population to be fully investigated and analyzed.
- We established applied downstream link prediction tasks based on the *HinSAGE* algorithm to demonstrate the efficient disease predictive model. This framework shows that the performance gained from EMR supports a sufficient significance to predict the outcome of an event within the patients and advocates for overall healthcare.

Furthermore, we proposed the method to build a graph database integrating EMR data. The EMR-embedded graph model was then applied to network learning using the *HinSAGE* algorithm. In this study, we illustrated the structure of the graph database and showed the query results to demonstrate the efficiency of the model. This research provides insights into physicians’ decision-making by predicting disease occurrence based on the performance of our implementation.

## Methods

**Figure 1:**
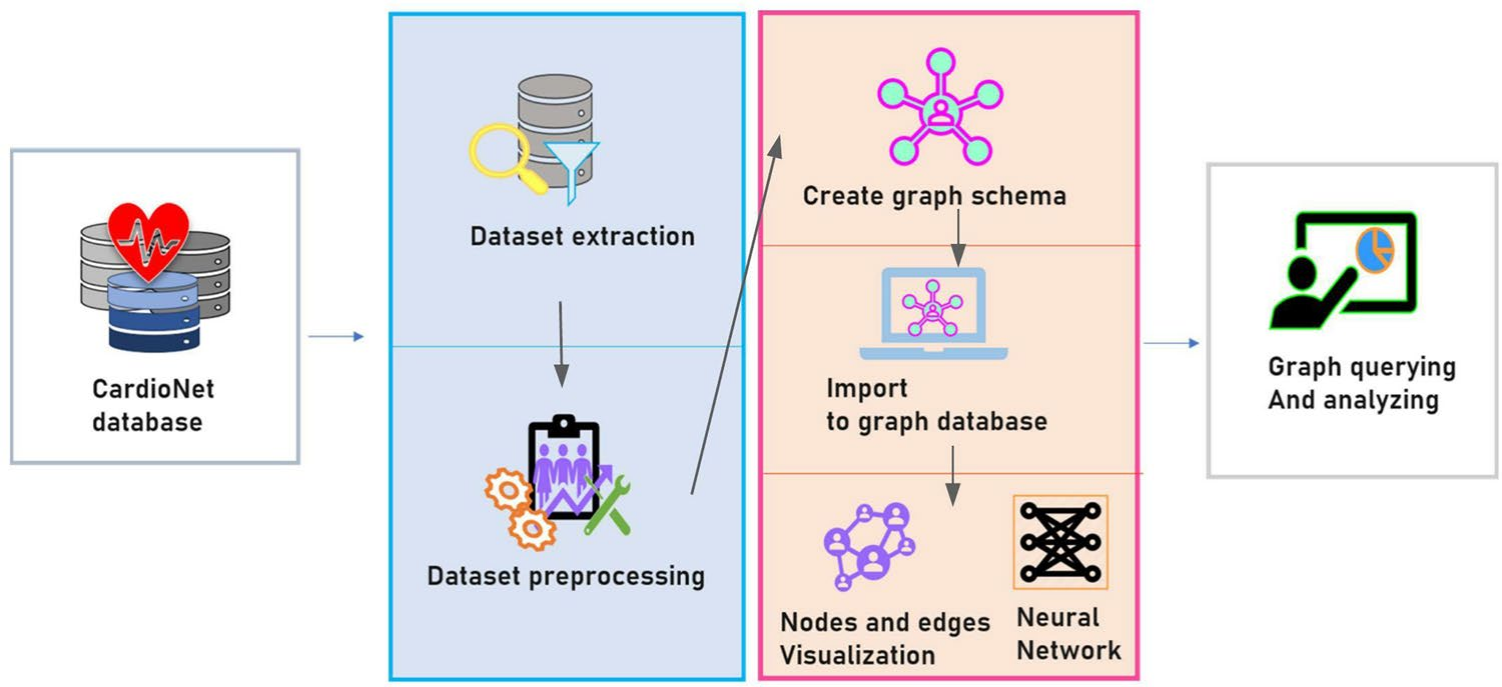
A pipeline for graph construction and graph neural network. The overall process was identical until the editing and manuscript step of the datasets. At this point, the graph schema production step differentiates.

An overview of the study methods are illustrated in Fig. 1. The datasets are mapped with the International Classification of Diseases, 10th Revision (ICD-10) code of the subject and imported with comma-separated value files. The extracted files were preprocessed using Python, then implemented for further analysis using *Neo4j* and the *Stellar graph* library. The graph network on *Neo4j* was based on Cypher query language. We used *Stellar graph*^33^ version 0.11.1 and *Neo4j*^34^ version 4.2.5.

In this study, two types of graph models were built based on different structures and analyzing purposes. The first graph was created on *Neo4j* for embedding patient records on EMR, which can then be used to efficiently visualize the patient journey and to find the associated data points with relatively easy query input. Our property graph was built with semantic mappings on shallow network embedding. Alternatively, the *Stellar graph* was used to create the second graph, where neural network prediction was performed.

### Data sources and the study cohort

The *CardioNet* database was composed of EMR data from a total of 53,841 patients with various cardiovascular diseases (CVD) within Seoul Asan Medical Center in South Korea. A patient population that had previously been admitted to the cardiology department, diagnosed with angina (ICD-10 code: I20), who never had been diagnosed with myocardial infarction (MI), stroke, and heart failure were selected. Inpatients alongside data from emergency-room encounters between January 1st, 2000, and December 31st, 2016 were included in the patient cohort; Outpatients were excluded.

**Figure 2:**
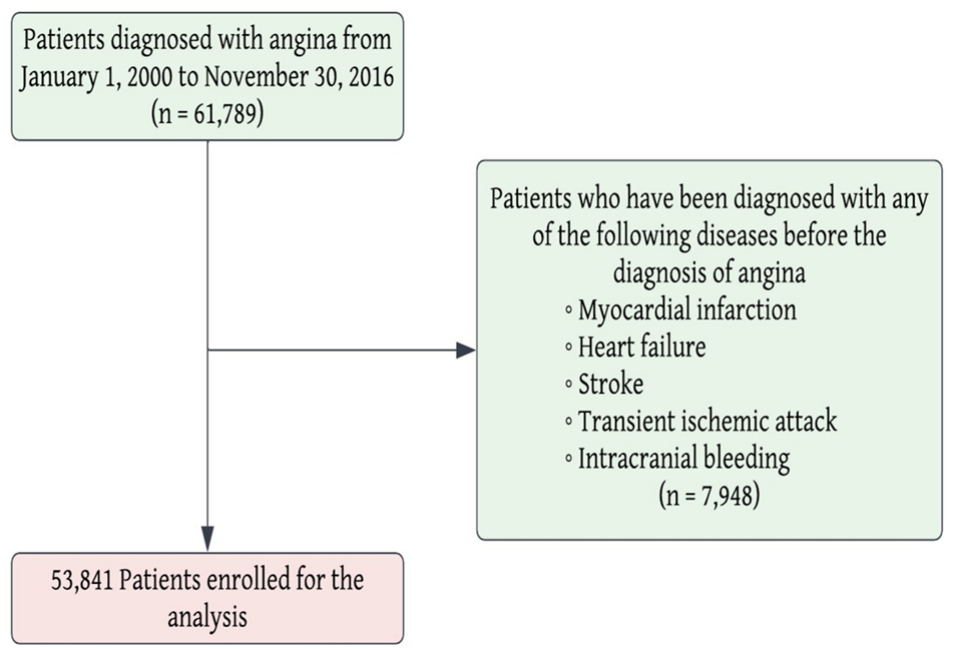
Flow chart of the patients included in the study.

Diagnosis, laboratory, echocardiography, physical, medication, surgery, visit and smoke datasets were extracted from *CardioNet* (Supplementary Fig. 1). The ICD-10^35^ was used to identify each patient’s health condition at admission. All patients are de-identified to the hospital's privacy rules, hence the individuals are given a unique patient ID which acts as a key linkage between the datasets. Individuals who were admitted with angina were observed for five years following an event to examine whether the patient suffered from further situations including death, MI, stroke, or heart failure as shown in Fig. 2. This study obtained approval and waived the written informed consent from the Institutional Review Boards of Asan Medical Center (No. 2021-0303). All experiments were performed in accordance with relevant guidelines and regulations.

### Graph construction with *Neo4j*

#### Graph schema construction

**Figure 3:**
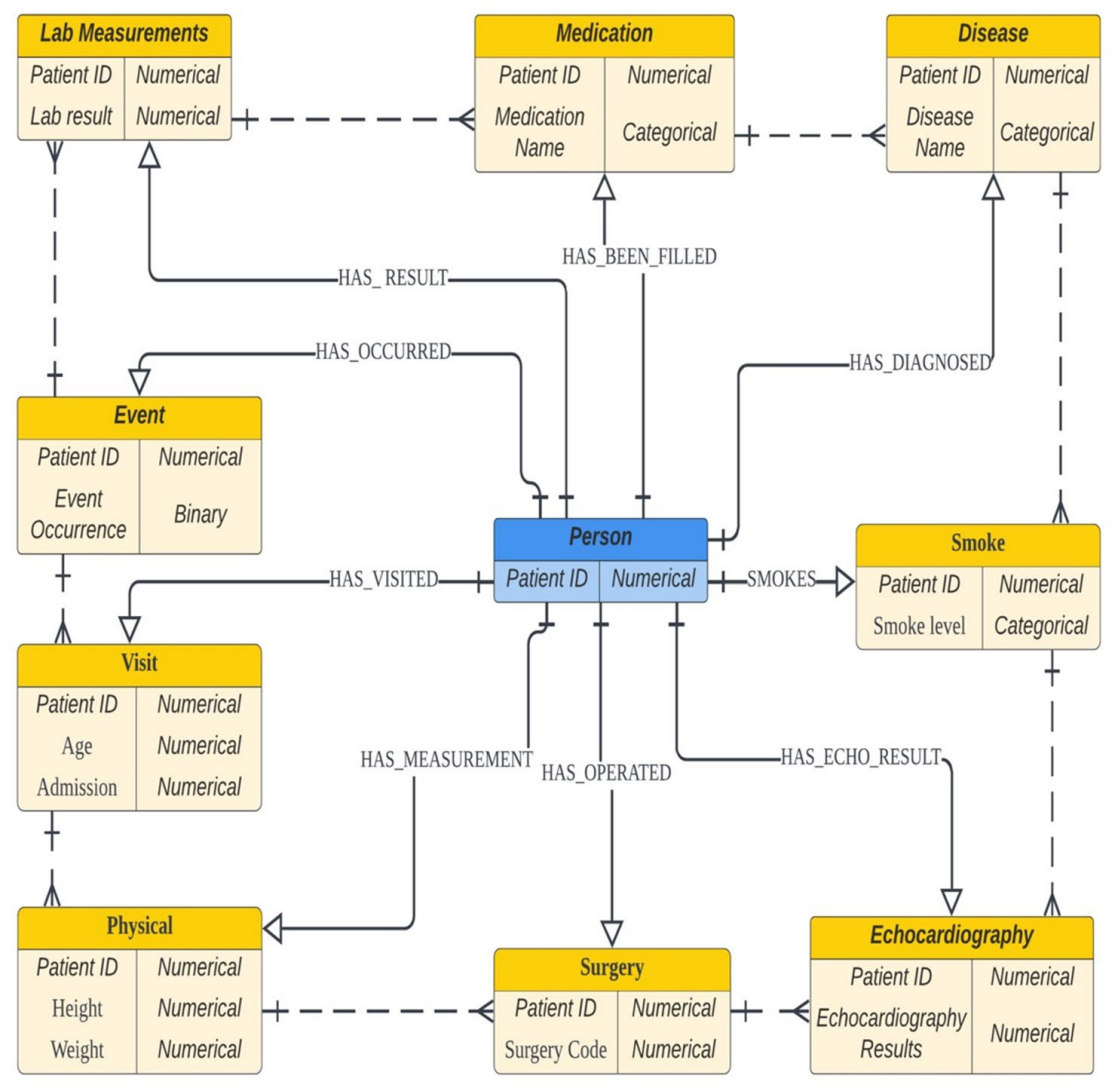
A finalized version of the schema for the patient entity graph. The solid line represents the bipartite relationship between the person and other nodes. In contrast, the dotted line between the nodes represents the possible connection that could be made during the query based on the user’s inquiry on the graph database.

Several different schemas were considered during the design phase, which included a process of trial and error of the structures until the most comprehensive schema was produced. To evaluate the best fit schema, we considered factors such as, whether the graph would be able to integrate different types of attributes that belonged to individual nodes and to represent different nodes. Also, every node has to somewhat relate to the patient to provide the attributes of each patient’s medical information. Consequently, the current model was able to satisfy the aforementioned criteria. During the designing process, we observed the following graph schemas: linear and circular, directed and undirected, fully connected and not fully connected, bipartite and non-bipartite, attributed and non-attributed, and weighted and unweighted graphs (Supplementary Data 2). A finalized version of the graph schema is illustrated in Fig. 3. Our graph model was constructed in a patient-centric method and multi-attributed. Further, it was multi-relational in terms of a heterogeneous set of edges, which form interactions within the network with its own edge labels. The graph was also created as a bipartite type, representing a not fully connected model. The relationship between the person node to other nodes were decided based on the consideration that a connection was made from the patient to every medical attribute the one received; your suggested bipartite schema is the optimal representation to retrieve the heterogeneous electronic medical records. See detailed information in Supplementary Data 2.

#### Entity and attribute selection

Ten types of entities were specified (Supplementary Data 3). All entity types contain two different forms of ID. Firstly, the unique patient ID was used for connecting the entities. Secondly, the default internal ID is generated by the *Neo4j* database. Supplementary Table 1 summarizes the node and node properties along with the data types of the properties for the graph model on *Neo4j*. The data types of the properties were decided based upon to achieve the maximum level of efficiency for visualization.

#### Relationships

To connect these entities, a total of nine different types of relationships were built for connecting the entities described in the previous section. The relationships were labeled according to the association between the entities, where the edge is destined. The starting node and ending node of the linkage were named with head and tail entities, respectively. All the node types, except the person node, begin from the person and are subsequently dispersed (Supplementary Table 2).

#### Building a graph database using cypher language

After cleaning the raw datasets and creating the schematic representation, the initiation of the graph building began with writing the code to import and represent the EMR datasets. The constraints of all node and edge types were applied to assert whether the patient ID property is unique among the node types. Indexes were then created with the following attributes to support the prediction of the node labels as a form of look-up method. Next, the nodes alongside their corresponding properties were imported with the dataset. The transactions with the periodic commit commander notably solved the deficiency in memory storage issues.

#### Graph visualization

While constructing the graph database, several factors relating to visualization were considered. The main purpose of the graph modeling on EMR was to detail the patient's journey through the graph in an effective, but simple method. The model patterns should therefore be able to provide insights at a quicker rate (please see Supplementary Fig. 2 for an example of a patient’s medical journey). Firstly, the colors and sizes of each entity were independently chosen so that each node type matched the corresponding edge types. Secondly, the thickness of the relationships connecting the data components was considered. A powerful benefit of graphs is the ability to show the linkages between the entities in the areas of interest. Lastly, the types of illustrated data attributes were carefully chosen to enhance the viewers’ instinctive visual understanding.

### Application: graph neural network with *Stellar graph*

#### Graph schema construction

It was decided that a heterogeneous, bipartite graph should be constructed with both node and edge attributes. Therefore two-node types were selected to represent each partition. Further types of datasets were integrated into the form of node attributes on each side. The outcome was included as the edge attribute separately which was specifically coded to define as an outcome column when formatting the edge table in the form of binary type. The outcome value was coded as 1 if the patient was admitted with angina, followed by the occurrence of death, MI, stroke, or heart failure for five years. On the other hand, if the angina patient was not diagnosed with either death, MI, stroke, or heart failure during the five years of follow-up, then the patient outcome was coded with 0. Therefore, the edge indicated 1 for a positive outcome, otherwise, 0 was displayed. Although the naming of two super nodes indicated patient and diagnosis, the tangible information portraying the data points in the graph indicated more than just the patient and their diagnosis.

#### Feature selection

The edge data frame for the *Stellar graph* embedding mechanisms was composed of three features: source, target, and outcome. Alternatively, the patient node’s data frame had 12 feature columns, while the diagnosis node’s data frame consisted of 147 feature columns. The details were recorded in Supplementary Data 4. Additionally, the data pre-processing step was identical to the pre-processing in Graph Construction with the *Neo4j* section.

#### Stellar graph construction

It is important that the index IDs that are unique to each row of the node’s data frame were used to connect the nodes in the graph with the edges. Therefore, in order to resolve the issues of duplicates forming, prefixes were added to all node indexes, against which the prefixed IDs of edges would eventually be matched. Subsequently, two different types of nodes that had been structured in data frames according to previously listed features were prepared. Eventually, the edge data will summarize each type of node relating to the relevant event outcome information. Overall, a graph model was built in combination with the nodes and edges data frame input.

#### Graph convolutional network: HinSAGE model

The advent of a relatively-new algorithm called, *HinSAGE*, a heterogeneous *GraphSAGE*^36^ algorithm, enables supervised graph embedding algorithms to maintain not only the topology of the dynamic graph but also the attributes of nodes and edges. *GraphSAGE* uses a generalized aggregation function inductively. In addition, the impact of applying features of nodes and edges plays a significant role in neural networks since features are the predicates of the subject of the study and thus should not be ignored.

The *HinSAGE*^37^ mechanism employs a two-step process of aggregating the representation of a target node. Firstly, by the neighboring node feature representations and by updating the embeddings on the final output of the nodes or graphs produced. To elaborate, we specifically chose *HinSAGE* because it was the optimal algorithm for applying datasets enriched in multi-attributed node features and relational heterogeneous large datasets. Additionally, the *HinSAGE* efficiently operates better than other multi-attributed algorithms, particularly for the outcome prediction with the outcome objective attached to the edge attributes.

Initially, in our development process, a graph object was created for a graph topology input. The two-node data frames and one-edge data frames were similarly embedded for heterogeneous graphs. The *HinSAGE* algorithm performed its samplings on the node neighbors in the graph structure. Following the creation of the graph, the resulting embeddings were input and split into the train and test sets, where the source, target, and labels were trained distinctively. Then, generators were created with specified node types and batch sizes for mapping the samplings. The two-layer *HinSAGE* model was then built with the input and output tensors exposing the sockets. Moreover, the estimator layer was added on top. Lastly, the *Keras* model was built for predictions and compiled by the custom optimizer, loss function for the minimization to fit the model, and metrics for evaluation (please see Supplementary Data 5 for full information on the model parameters in the experiment setting).

## Results

### Baseline characteristics of participants

Baseline clinical characteristics of 53,841 total patients between January 1st, 2000, and December 31st, 2016 were presented in Supplementary Table 3 as a result of the patient exclusion/inclusion process in Fig. 2. The total population was stratified by the event positive and the event negative (the event outcome was coded as 1 for death, MI, or heart failure occurrence and 0 for no occurrence). In these cases, the mean age was slightly older for the positive population compared to the negatives. Also, regarding the underlying symptoms, higher rates were observed in the positive group than in the negative group (Supplementary Table 3).

### Network findings and visualization with queries

The *CardioNet* database was used to build a patient-centric graph database. After the preprocessing steps on the cohort, as previously described a total of 53,841 patients was obtained from 572,811 CVD patients and used to build two types of networks alongside learning the graph neural network. The entities and quadruplets counted on the Neo4j graph database were 492,886 and 439,045 respectively for displaying 10 node types and 9 edge types, respectively (Supplementary Fig. 4). Alternatively, the nodes and relationship figures on the *Stellar graph* were 107,682 for nodes and 53,841 for edges (Supplementary Fig. 3). The node types for representing patients and diagnosis showed feature information that the vector type is float 32 with lengths of 12 and 147, respectively. However, because there is a weight added to the edges in the graph model, the feature length shows 1 under the edge information. The final constructed version of the *Stellar graph* was an undirected multigraph type. The numbers of the *Ste*llar graph nodes and edges are smaller compared to the *Neo4j* network because there is limited target outcome information available relating to both the node and edge types. Additionally, the size of the feature input was different between the two networks considering the objective of each network.

After creating the graph database, a single patient’s journey was sub-filtered (Supplementary Fig. 2). Firstly, a relationship type of interest was assigned before filters were set with specific medication names. These were filtered by applying the DISTINCT function to exclude any duplicated patient results, which provided the total query results. Each node type contains its associated edge types, which are illustrated by the matching colors. The graph contains patient data relating to a de-identified patient ID of 107411, who has been diagnosed with hypertension, operated on with diagnostic ultrasound, smoking level of 1, resulted in laboratory blood with 94.0, has echo result of − 1.0, and physical measurement of 53.55. Moreover, the patient has been prescribed statin medication; and suffered from an event at the age of 67 when visiting the hospital. The details of node properties are restricted, and the other properties are hidden within a network until the user either visualizes them or modify the display mode of the node’s property (Supplementary Fig. 2).

Next, several questions were queried in Cypher codes which were used to analyze the performance of the graph database. The query results are potentially either in the graph structure or tables, depending on the user’s query question. By utilizing the EMR network, the queries and their results, alongside the time taken to complete each query can be depicted in a table format (Supplementary Fig. 5). It depicts the speed and effectiveness of a graph database that can pull out the results from only a few lines of code. Moreover, Supplementary Figs. 6 and 7 display the graphical query results gathered from query questions as 1 and 4 were depicted in Supplementary Fig. 5. Indeed, Supplementary Fig. 6 illustrates the zoomed-in result of the three filtered patients, who were treated with Statin medication. Moreover, we wanted to visualize the patients with a previous diagnosis and an event outcome of 1 (Supplementary Fig. 7).

### *HinSAGE* model evaluation and comparisons

A supervised link attribute inference problem appeared on two different node types, the attributes, along with the edge binary attributes of 0 and 1 for the prediction targets. The performance of the training history from the finalized *HinSAGE* model selected was exhibited in Supplementary Fig. 8. During 30 epochs of the model training, the accuracy plot shows comparable performance for both validation and training datasets. The accuracy plot reveals a training accuracy and a validation accuracy of 0.73 and 0.67, respectively (Supplementary Fig. 8a). Moreover, the general trend for the training loss plot shows linearity beginning at epoch five, whereas there are up and down variations for validation loss resulting from occasions as the epoch reaches the end (Supplementary Fig. 8b). Further, the distribution of true and predicted outcomes for the test set is shown in Supplementary Fig. 8c. In this plot, the true outcome for zero has more than ten times the value of ones meaning there is much more than ten times greater the number of events that did not occur than events that occurred. Expectedly, the predicted values for the outcome follow the same trend, presenting an increased number of counts towards the event that did not happen than did. The plot was drawn using the Matplotlib library on python pandas.

**Figure 4:**
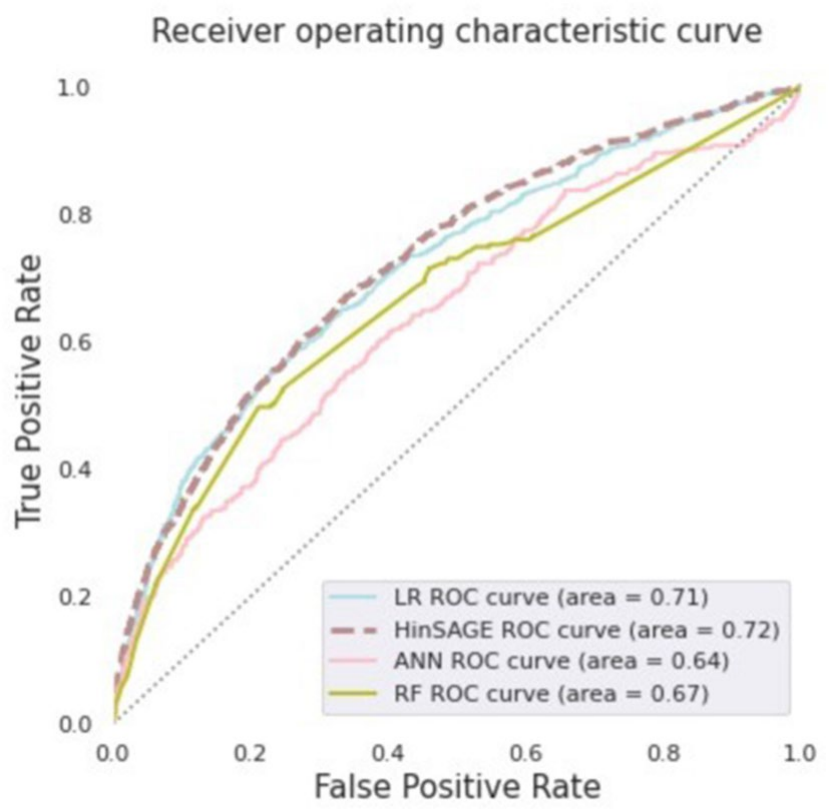
Receiver operating characteristic curves of comparison models in predicting patient disease occurrence.

We compared our *HinSAGE* model to three prominent machine learning methods: random forest^38^ (RF), logistic regression^39,40^ (LR), and artificial neural network^41^ (ANN) models. The outcomes for comparative evaluation are shown in Table 1. In this study, we used the AUROC and AUPRC scores alongside the 95 percent confidence interval range to evaluate the model performance in predicting the patient disease outcome. The result displays that the *HinSAGE* model outperforms the AUROC and AUPRC scores, achieving 0.72 and 0.15, respectively with 0.71 to 0.74 CI and 0.14 to 0.16 CI ranges. Further, AUROC scores demonstrate that there is no significant difference between the comparison models and the *HinSAGE* model. Figure 4 demonstrates the receiver operating characteristic curves for all prediction models, where the x-axis and y-axis represent the false positive rate (1-specificity) and true positive rate (sensitivity). The ROC curves clearly show that the prediction was successful of the models (Fig. 4).

**Table 1:** Comparison of different model performances for baseline models and *HinSAGE*.

	AUROC (95% CI)	AUPRC (95% CI)
RF	0.67 [0.63, 0.70]	0.11 [0.09, 0.12]
ANN	0.64 [0.60, 0.68]	0.11 [0.09, 0.12]
HinSAGE	0.72 [0.71, 0.74]	0.15 [0.14, 0.16]
LR	0.71 [0.67, 0.74]	0.13 [0.12, 0.15]

## Discussion

In this study, we provided the methodologies for building a patient-centric schema on highly heterogeneous and relational EMR datasets unique to 53,841 patient individuals. A total of ten types of entities and nine relationships with the node properties in particular for each node type were created. We then presented a multi-attributed graph database based on the schema, and the performance was shown with eight successful query results. Second, we provided methodologies for building a downstream graph model for disease outcome prediction. We presented two node types, one with twelve features and another with one-hundred forty-seven features. We then applied the *HinSAGE* algorithm to evaluate the model as a supervised link prediction problem. As a result, the *HinSAGE* model’s performance led to 0.72 and 0.15 AUROC and AUPRC scores.

Firstly, the former type of patient-centric model offers a comprehensive analysis. Similarly, our graph database reveals the significance of the attributes attached to nodes or edges. Furthermore, by adopting various color palettes and sizes to the different node and edge types, the user’s adaptability in perceiving the information supplied in the network was increased. Moreover, the querying result effectively showed that a strategically designed graph database provides insights within a short period of time, for both the experts and all the non-experts. Next, for the latter type of graph model, *HinSAGE* was particularly chosen over other methods because it was effective for heterogeneous data with rich node attributes. Also, we developed a model in consideration of edge weights to provide a link inference task suited to a bipartite patient model with node and edge attributes. The result demonstrates that the inclusion of outcome to edge weights increases the model accuracy since the attached stacked layers of outcomes link the full association information, which will support the overall prediction. Additionally, the *HinSAGE* model plot indicates that the learning process on the training data has been successful.

Lastly, during the evaluation process, the *HinSAGE* model achieved the highest performance scores compared to other methods. The AUC scores in Table 1 show no significant difference between the models, indicating the performance at distinguishing between the occurrence classes of the event is comparable to the baseline methods. To elaborate, because the problem itself of predicting the medical outcome is very difficult, we initially aimed at achieving a comparable score for the *HinSAGE* model as other baseline models’ performances. Hence, despite the formal reasons for achieving not much higher AUC, utilizing EMR data in research opportunities offers many other beneficial aspects.

Therefore, we demonstrate that the prediction is beneficial when graphical topology and attributes are learned together rather than solely obtaining the features. Clearly, these results verify the significance of the graph neural network, particularly indicating that the *HinSAGE* model is competent enough to learn patients' data and analyze the disease prediction from the EMR data when compared to other fundamental learning methods. Importantly, the mechanisms exist for the conventional machine learning methods, which implement EMR records. However, it is vastly easier to utilize the built-in graph representation learning method, which enables nonprofessionals to understand the importing process for both the EMR structure and the deep learning steps. Therefore, despite the complex knowledge requirements for deep learning computation, our pipeline is easily manageable and will support the non-technical expertise throughout the overall processes, without requiring prior knowledge of the field. Consequently, this serves to provide a deeper understanding of predictive and precision medicine. For instance, our graph database is adequate to support efficient knowledge extraction for medical research, and self-diagnosis or self-education services for patients. It also provides graph learning along with the graph learning framework for supporting clinicians in decision-making. In regards to further developments, adding datasets from other countries may support the confirmation of universality, hence, various data sources can be applied in the framework to improve the model’s representation. Also, future works may integrate additional node types, node attributes, and edge weights to further increase the accuracy in the performance of the inpatient disease predictions.

## Conclusions

Our study suggested a visualization method for EMR using two visualization frameworks and how they are built and applied. The graph database was performed to query at efficient runtime and is easily modifiable in real time. Further, benefiting from the graph neural network mechanisms, this work presented a favorable framework compared to previous baseline methods: The *HinSAGE* was implemented to efficiently predict CVD event outcomes using supervised link prediction learning. Our work demonstrated that graph databases and graph neural networks are great options for building high-dimensional models, for use in the predictive analysis of heart disease treatments.

## Supplementary Information

Supplementary Information.

## Data Availability

Supporting data are available from Asan Medical Center, and due to ethical concerns and confidentiality agreements, data are available upon reasonable request.
