# Supplementary material for: Heterogeneous graph construction and HinSAGE learning from electronic medical records
Source: Sci Rep. 2022 Dec 7;12:21152. doi: 10.1038/s41598-022-25693-2 (PMC9729175; doi:10.1038/s41598-022-25693-2)
Supplement: Supplementary file 1 — Supplementary Information. [file 41598_2022_25693_MOESM1_ESM.pdf]

Heterogeneous graph construction and HinSAGE learning from electronic medical records

Ha Na Cho[1], Imjin Ahn[2], Hansle Gwon[2], Hee Jun Kang[1], Yunha Kim[2], Hyeram Seo[2] , Heejung Choi[2], Minkyoung Kim[2], Jiye Han[2], Gaeun Kee[1], Tae Joon Jun[3,+] & Young-Hak Kim[1,+]

[1]Division of Cardiology, Department of Internal Medicine, Asan Medical Center, University of Ulsan College of Medicine, 88, Olympicro 43gil, Songpagu, 05505, Seoul, Republic of Korea

[2]Department of Medical Science, Asan Medical Institute of Convergence Science and Technology, Asan Medical Center, University of Ulsan College of Medicine, 88, Olympicro 43 gil, Sonpagu, 05505, Seoul, Republic of Korea

[3]Big Data Research Center, Asan Institute for Life Sciences, Asan Medical Center, 88, Olympicro 43gil, Songpagu, 05505, Seoul, Republic of Korea

+These authors contributed equally to this work

Corresponding author:

Young-Hak Kim, MD, PhD

Division of Cardiology, University of Ulsan College of Medicine

88, Olympic-ro 43 gil, Songpa-gu, Seoul 05505, Korea

Phone: 82 2 301 0 3955

Email: mdyhkim@amc.seoul.kr

Supplementary Fig. 1. Datasets extracted from the CardioNet database.

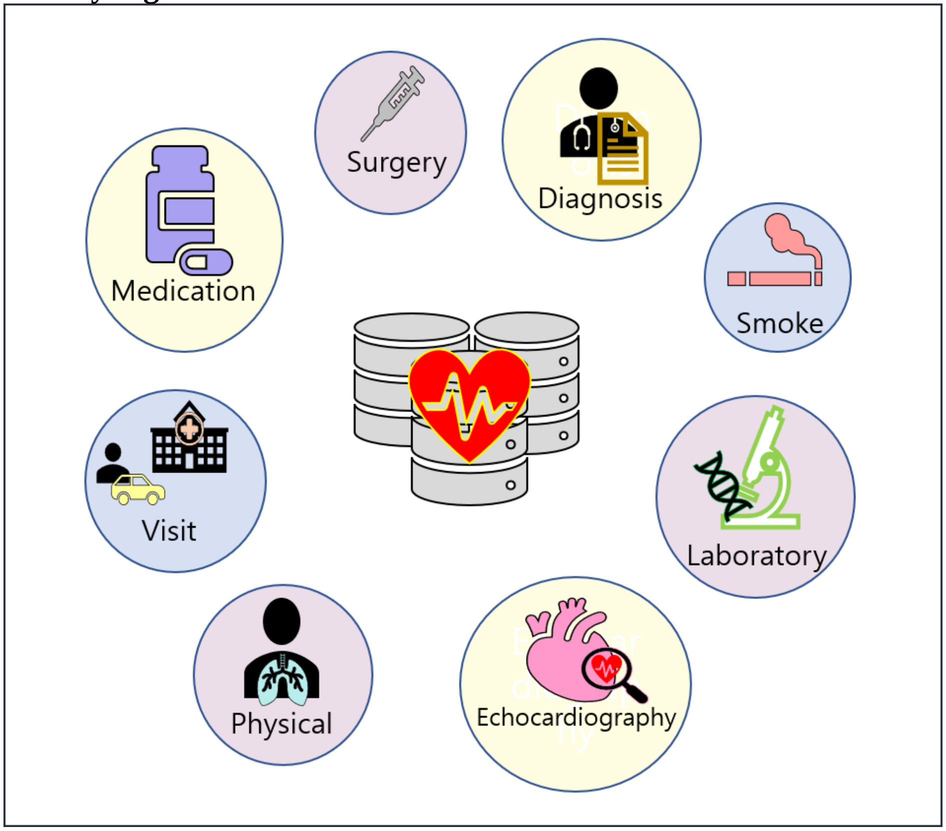

Supplementary Data 1. Cohort preparation and data preprocessing.
Given the CardioNet datasets, patients of the study subject were initially defined by filtering the population diagnosed with angina (ICD-10 code: I20), which was defined as 'indexDT'. Next, the study outcome during the event period was defined. The event period was inserted between 0 to 1825 days, where any of the null values for the event period was filled with NaNs. Subsequently, death, MI (ICD-10 code: I21, I22, I23), stroke (ICD-10 code: I60-I69), and heart failure (ICD-10 code: I42, I43, I50) were extracted for the five years following the primary angina occurrence. The predictive factors were mainly selected by the support of cardiologists' assistance in deciding the variable's importance and suitability. Figure 2 presents a flow chart describing patients included in the study.

Supplementary Data 2. Graph Schema Construction.

A schema-building process occurred prior to importing the datasets into the database. The domain of the graph schema reflects how the entities are related to each other, indicating any slight modification in the layout of the schema will provide different outcomes depending on its schema.

While observing different schemas, we found that a linear graph cannot create efficient relationships with other nodes, thus, we used a circular structure to efficiently interact with nodes of more than two. Next, a directed edge format was selected, instead of undirected edges, to particularly describe the individual relationship connecting to specific nodes. Then, we wanted to create not a fully connected schema to provide a patient-centric graph, thereby, choosing a bipartite structure. For instance, It is costly to implement a fully connected network and requires an increase in query time as the number of connections between nodes and edges grows. Lastly, the graph needed to contain feature information with patients detailed medical records and the outcome values attached to perform the prediction, therefore, an attribute and weight graph schema was built.

Thus, significant factors were used in the prediction stage, which contributed to the event outcome. The event node illustrating the subject of the study was created as a separate node so that the query output can be focused on the properties attached to the event entity. This allowed the association between the subject outcome with other predictive properties to be evaluated. The detailed steps included deleting and merging entities, creating specific edges between the entities, aligning the node properties to edge properties, and simplifying the data structure.

Our graph model was constructed in a patient-centric method, where each patient was designed to be the center of each graph. Hence, patient individuals created a unique graph of their own containing the total medical records of a visit. In addition, the network was multi-attributed meaning each entity type has its corresponding set of attributes or features. Further, it was multi-relational in terms of a heterogeneous set of edges, which form interactions within the network with its own edge labels. The graph was also created as a bipartite type, where the sets of vertices can be disjointed into two groups, representing a not fully connected model.

Supplementary Data. 3. Entity and Attribute Selection.

Event: Event has two values for identifying the occurrence of the outcome, 0 indicates no event, and 1 is the occurrence of an event.

Person: The person entity is represented in the core of each patient's graph model, whereby all additional types of entities were linked to the individual. The person entity solely represents the unique patient ID.

Echocardiography: Echocardiography results provide several items of information for the measurement of the heart's pumping strength. The criteria for the measurements are right information rhythm, systolic left ventricular internal diameter, diastolic left ventricular internal diameter, systolic left ventricular wall stress, diastolic left ventricular wall stress, systolic left ventricular summit, diastolic left ventricular summit, left atrium size, aorta diameter, left systolic volume, left diastolic volume, left ventricular blood velocity1, left ventricular blood velocity2, blood velocity ratio, velocity-time, mitral regurgitation grade, aorta velocity, tricuspid valve grade, tricuspid velocity, tricuspid reflux max velocity, pressure difference, plax and psax (11-46), echo window, left ventricular mass index, septal velocity1, septal velocity2, left ventricle ratio, wall motion index, systolic blood pressure, diastolic blood pressure, systolic septal velocity, left ventricular ejection fraction, left ventricular mass.

Surgery: The Surgery entity consists of the surgical procedure code provided at each patient visit.

Smoke: The smoke data are designated as 1,2, or 3 depending on the level of a patient's smoking status. Here, 1 represents smoking, 2 as never smoked, and 3 equates to having stopped smoking.

Visit: Each patient's visit was recorded alongside the information of age, duration in days, and account number.

Physical: Measure entity contains gender, height, weight, systolic and diastolic blood pressure, pulse rate, and respiration rate.

Disease: Patients are admitted with the ICD-10 code, which illustrates the designated diagnosis code acquired. The disease entity displays the diagnosis of each patient.

Medication: The generic name and the ingredients of each drug prescribed are provided.

Laboratory: The laboratory exam name and exam results are provided.

Supplementary Table 1. List of nodes and node properties.

Node	Node properties	Types
Person	ID	Numerical
Event	ID, event	Binary
Disease	ID, disease name	Categorical
Echocardiography	ID, right information rhythm, systolic left ventricular internal diameter, diastolic left ventricular internal diameter, systolic left ventricular wall stress, diastolic left ventricular wall stress, systolic left ventricular summit, diastolic left ventricular summit, left atrium size, aorta diameter, left systolic volume, left diastolic volume, left ventricular blood velocity1, left ventricular blood velocity2, blood velocity ratio , velocity time, mitral regurgitation grade, aorta velocity, tricuspid valve grade, tricuspid velocity, tricuspid reflux max velocity , pressure difference, plax and psax (11-46), echo window, left ventricular mass index, septal velocity1, septal velocity2, left ventricle ratio, wall motion index, systolic blood pressure, diastolic blood pressure, systolic septal velocity, left ventricular ejection fraction, left ventricular mass	Numerical
Physical	ID, gender, height, weight, systolic blood pressure, diastolic blood pressure, respiration rate, pulse rate	Numerical
Medication	ID, medication name	Categorical
Smoke	ID, smoke	Categorical
Surgery	ID, surgery code	Numerical
Visit	ID, age, days hospitalized, admission count	Numerical
Laboratory	ID, lab result, lab exam type	Categorical<br>Numerical

Supplementary Table 2. List of relationships, types, and descriptions.

Relationships	Relationship types	Description
Person to Event	HAS_OCCURRED	Event outcome displayed
Person to Disease	HAS_DIAGNOSED	Disease names displayed
Person to Echocardiography	HAS_ECHO_RESULT	Echocardiography results displayed
Person to Physical	HAS_MEASUREMENT	Physical measurements displayed
Person to Medication	HAS_BEEN_FILLED	Treated medication displayed
Person to Smoke	SMOKES	Smoking level displayed
Person to Surgery	HAS_OPERATED	Operation code displayed
Person to Visit	HAS_VISITED	Admission information displayed
Person to Laboratory	HAS_RESULT	Laboratory exam and results displayed

Supplementary Fig. 2. An example of a patient's medical journey as a graph database. The visit node in green represents the age, the smoke node in red represents the level of smoking, the surgery node in gray represents the surgery code that reflects the patient's number, the event node in navy represents the event occurrence, a physical node in purple represents weight, the echocardiography node in pink represents the tricuspid valve regurgitation grade, the diagnose code in yellow represents the diagnosis name and the medication node in orange represents the medication name.

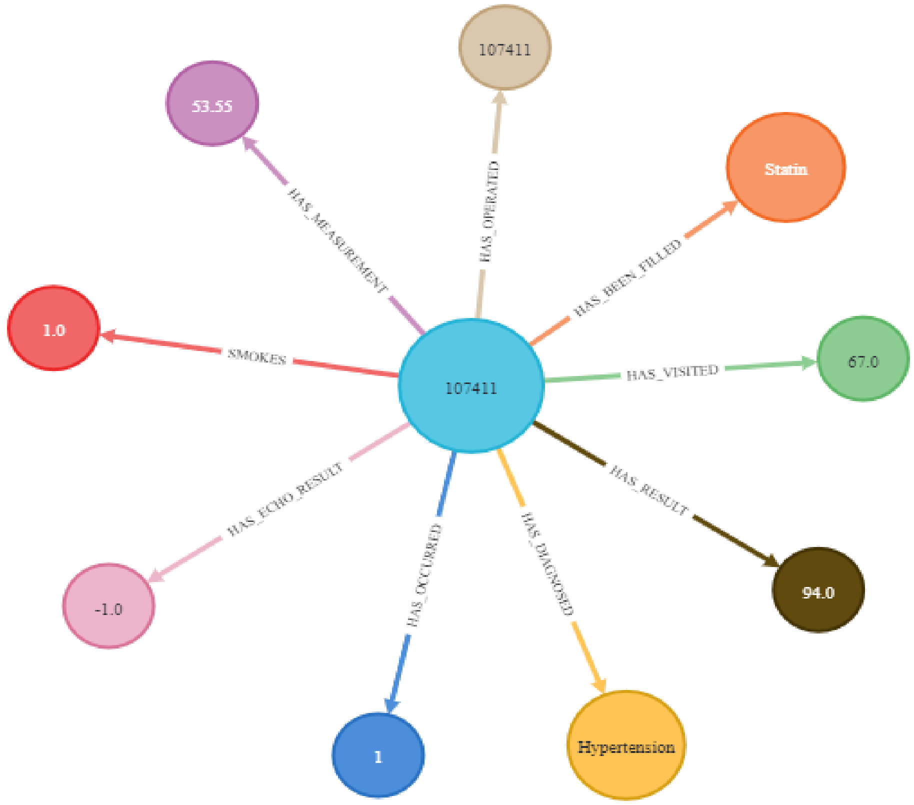

Supplementary Data. 4. Feature selection and data pre-processing.

The attributes for patient nodes contained general patient information at the visit including, age, gender, duration of hospitalization for count and days, year, height, smoking status, weight, pressure rate, respiration rate, and systolic and diastolic blood pressure. Moreover, the diagnosis node attributes included attributes from diagnosis, medication, laboratory, echocardiography measurements, and surgery information. The details are recorded as follows:

Diagnosis: arrhythmia, atrial fibrillation, cancer, chronic ischemic heart disease, chronic lung disease, diabetes mellitus, dyslipidemia, hypertension, liver disease, peripheral arterial disease, pulmonary embolism, renal disease, valvular heart disease.

Medication: Angiotensin-converting enzyme inhibitor, adenosine diphosphate receptor inhibitor, angiotensin receptor blockers, aldosterone antagonist, allopurinol, an alpha-glucosidase inhibitor, aspirin, beta-blockers, calcium channel blocker, calcium channel blocker, non-dihydropyridine, cilostazol, a dipeptidyl peptidase-4 inhibitor, direct oral anticoagulant, diuretic-loop, diuretic-thiazide, diuretic-thiazide-like, insulin, meglitinide, metformin, nitrate, other antianginals, other lipid-lowering, Protease-activated receptors agonist, sodium-glucose cotransporter 2 inhibitor, statin, sulfonylurea, and warfarin.

Measurement and Laboratory: ALT, AST, albumin, alkaline phosphatase, antithrombin III, apolipoprotein B, apolipoprotein A1, BNP, BUN, bilirubin, CK, CRP, calcium, chloride, cholesterol, creatinine, d-dimer, ESR, FDP, ferritin, fibrinogen, glucose, HDL-cholesterol, Hb, homocysteine, LDL-cholesterol, lp, pt, phosphorus, platelet, potassium, protein, sodium, triglyceride, uric acid, WBC, ptt activation, eGFR, eGFR, hsCRP.

Cardiography: systolic left ventricular internal diameter, diastolic left ventricular internal diameter, systolic left ventricular summit, systolic left ventricular wall stress, diastolic left ventricular wall stress, diastolic left ventricular summit, left atrium size, aorta diameter, left diastolic volume, echo window, left ventricular mass index, left ventricle ratio, left ventricular ejection fraction, left ventricular mass, left systolic volume, blood velocity ratio, tricuspid reflux max velocity.

Operation: Removal of coronary artery obstruction and insertion of stent, Other operations on vessels, Diagnostic procedures on lung and bronchus, puncture of vessel, Extracorporeal circulation and procedures auxiliary to heart surgery, Other shunt or vascular bypass, repair of vessels, bypass anastomosis, operation on thorax, diagnostic on chest wall, replacement of heart valve, insertion, lobectomy, endovascular repair, implementation, resection, local excision, pericardiectomy, incision of chest wall, other operation on lung, excision on larynx, open heart valvuloplasty, revision of vascular procedure, insertion of pacemaker, diagnostic on heart and pericardium, cardiotomy, pericardiocentesis, temporary tracheostomy, vessel incision, interruption of vena cava, other operation on pericardium, operation on structures adjacent to valves, operation on septa of heart, endarterectomy, diagnostic on trachea, repair of atrial, excision of vessels, surgical collapse of lung, surgical occlusion of vessels, ligation of veins, complete pneumonectomy, excision of chest wall, repair of chest wall, repair of atrial septa, pleurectomy, operation on diaphragm, excision on tissue of mediastinum, suture of vessel, repair of plastic operation on trachea, repair of pericardium and heart.

Supplementary Data. 5. Experimental settings.

The evaluation of the model performance was on predicting the link attribute for the event occurrence. The model was in python using the stellar graph library's HinSAGE model and the scikit-learn package. The experiments were conducted with various combinations of hyperparameters until the optimal combinations for the best fit model in an accuracy score were achieved. The edges into training and test sets in our dataset were split by a ratio of .6:.4. At the beginning of the experiment, a random set was applied to provide reproducible results. The size of the epochs trained was 30 with both no dropout and no bias and a batch size of 200. We then created the HinSAGE model object. The HinSAGE model was built with a stack of layers, where the hidden layers measured 2 by 2.

Further, the model was trained with the HinSAGE link generator, which integrated the edge graph structure and the subject of event outcome, while attaching them to the edge properties. Furthermore, it indicated the head node types of patients and diagnoses. During the stacking process, and to clearly limit the number of layers in the model, the length of the layers was assigned to equal the length size of the number of samples. Following the HinSAGE layer parameters, the final estimator layer for the prediction was set to link the regression to concatenate them for forming an edge representation. Moreover, the predictions and the sigmoid activation layer were added to transform the node properties in the HinSAGE model into the Keras model. After the model layers were created and representation methods arguments configured, the Keras model was compiled to train with 10 workers assistants, where we used the Adam optimizer with a learning rate of 0.01, binary cross-entropy loss, and the metrics to evaluate set-to accuracy.

The aforementioned hyper-parameter setting described our optimal combinations for the final result. When considering the hyperparameters, the number of epochs, number of samples, HinSAGE model layer size, dropouts, metrics for model evaluation and losses, the learning rate for the optimizer, and the logits for edge embeddings all affected our model selection.

Additionally, to compensate for the under-represented class of the unbalanced outcome label, the distribution of the class labels was manually controlled by applying the class weights parameter while training the model. The class weight was set to equally balance the class labels of train data and mapped into unique values to fit in the dictionary format.

Supplementary Table 3. Baseline clinical characteristics of the patient population by event outcome.

	All population	Negative	Positive
	(n=53,841)	(n=50,765)	(n=3076)
Age (years), mean (s.d.)	61.1 ± 10.7	60.8 ± 10.6	65.0 ± 10.6
Gender, n (%)			
Male	34,353 (61.6)	32,304 (61.4)	2,049 (66.1)
Female	19488 (38.4)	18,461 (38.6)	1,027 (33.9)
Body mass index (kg/m2), mean (s.d.)	24.7 ± 3.1	24.8 ± 3.1	24.1 ± 3.5
Systolic blood pressure, n (%)	126.3 (18.6)	126.2 (18.4)	128.6 (21.4)
Hypertension, n (%)	16,705 (30.0)	15,331 (29.1)	1,374 (44.3)
Diabetic mellitus, n (%)	8,737 (15.7)	7,849 (14.9)	888 (28.7)
Renal disease, n (%)	2,252 (4.0)	1,767 (3.4)	485 (15.7)
Atrial fibrillation, n (%)	1,069 (1.9)	904 (1.7)	165 (5.3)
Cancer, n (%)	3,640 (6.5)	3,260 (6.2)	380 (12.3)
Chronic lung disease, n (%)	1,568 (2.8)	1400 (2.7)	168 (5.4)
Liver disease, n (%)	1,498 (2.7)	1,365 (2.6)	133 (4.3)
Peripheral arterial disease, n (%)	1,095 (2.0)	937 (1.8)	158 (5.1)
Valvular heart disease, n (%)	1,103 (2.0)	938 (1.8)	165 (5.3)
Pulmonary embolism, n (%)	186 (0.3)	143 (0.3)	43 (1.4)
Left ventricle ejection fraction, mean (s.d.)	59.5 ± 8.8	60.3 ± 8.3	54.7 ± 12.5
Left ventricle mass (g), mean (s.d.)	183.5 ± 57.1	181.6 ± 55.8	207.5 ± 67.2
E/A ratio, mean (s.d.)	12.0 ± 3.9	11.9 ± 3.9	13.7 ± 4.3

Supplementary Fig. 3. Summary of a Stellar graph description.

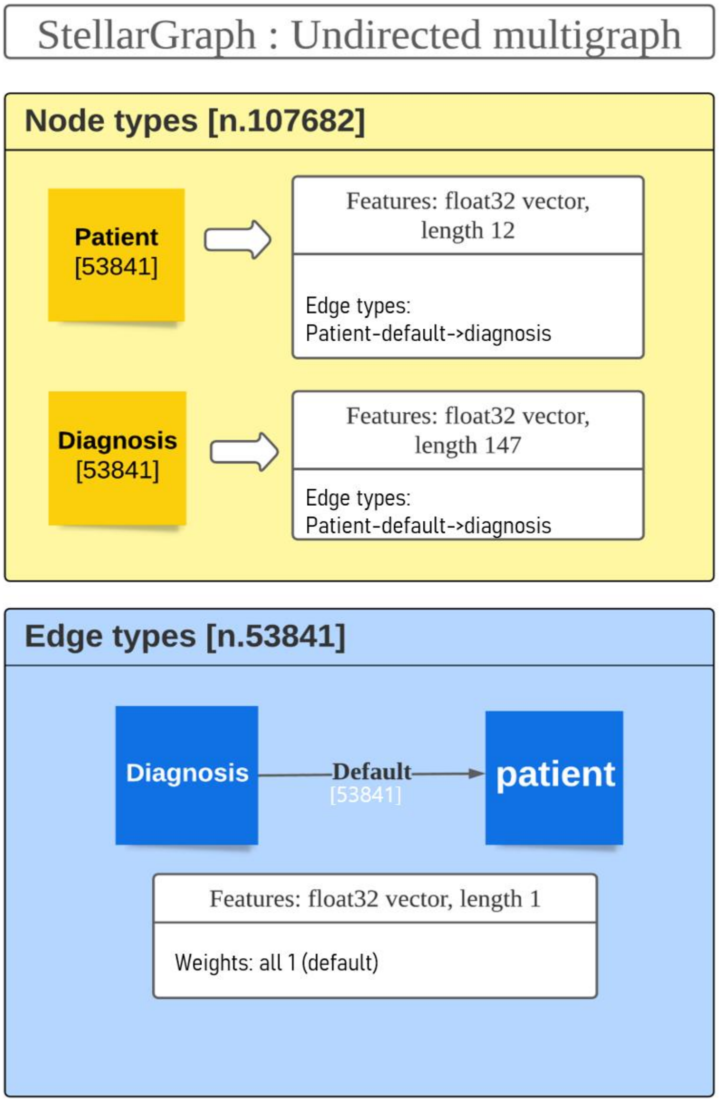

Supplementary Fig. 4. Summary of node labels and relationship types.

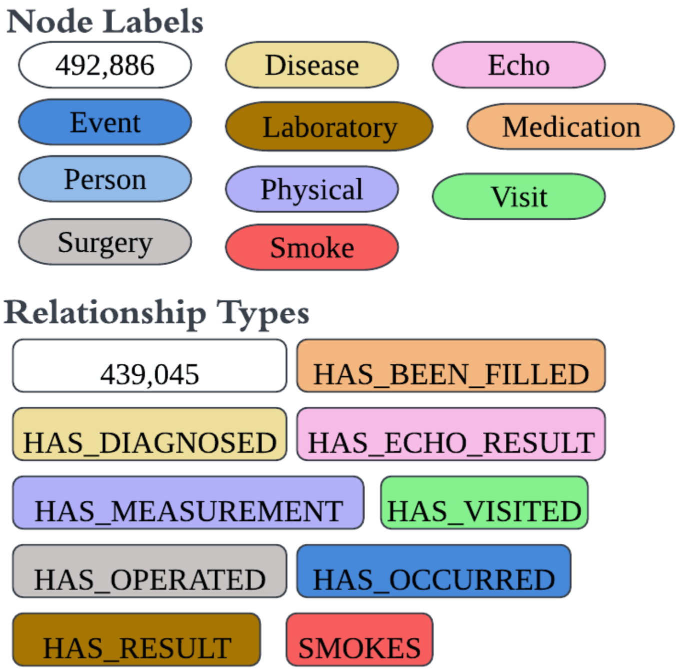

Supplementary Fig. 5. Neo4J query questions and results. The questions relating to such as finding the number of patients treated with a particular drug, or diagnosed with a certain disease, alongside the total number of patients who have or have not experienced an event, distribution of average age, distribution of diagnosis, and the order of highly occurring disease and drugs were queried.

	Question	Cypher Query	Result	Time completed (ms)
1	Number of patients treated with Statin medication	MATCH (p)-[r:HAS_BEEN_FILLED]-<br>>(m:Medication)<br>WHERE toLower(m.odcdnm) CONTAINS<br>"statin"<br>RETURN count(DISTINCT((p.id)))	10205	48
2	Number of patients treated with hypertension	MATCH (p)-[r:HAS_DIAGNOSED]-<br>>(d:Disease)<br>WHERE toLower(d.dicdnm)<br>CONTAINS 'hypertension'<br>RETURN count(distinct(p))	13108	25
3	Number of patients that has an event of 0 outcome	MATCH (p)-[r: HAS_OCCURRED]->(e)<br>WHERE e.event = '0'<br>RETURN COUNT(DISTINCT(p))	40551	41
4	Number of patients that has an event of 1 outcome	MATCH (p)-[r: HAS_OCCURRED]->(e)<br>WHERE e.event = '1'<br>RETURN COUNT(DISTINCT(p))	2033	22
5	Number of patients diagnosed with Chronic disease	MATCH (p)-[r:HAS_DIAGNOSED]-<br>>(d:Disease)<br>WHERE d.dicdnm CONTAINS 'Chronic '<br>RETURN count(distinct(p))	5165	18
6	Top five highly occurred disease among patients who had a positive event occurrence (event=1)	MATCH (p:Person)-[r:HAS_DIAGNOSED]-<br>>(d:Disease)<br>WHERE exists ((p)-[:HAS_OCCURRED]-><br>(:Event {event:'1'}))<br>RETURN d.dicdnm AS disease, count(*) AS<br>freq<br>ORDER BY freq DESC LIMIT 5	1. Hypertension<br>2. Renal disease<br>3. Chronic ischemic heart<br>disease<br>4. Diabetes mellitus<br>5. Valvular heart disease	77
7	Top five drugs treated d among patients who had a positive event occurrence (event=1)	MATCH (p:Person)-[r:HAS_BEEN_FILLED]-><br>(m: Medication)<br>WHERE exists ((p)-[:HAS_OCCURRED]-><br>(: Event {event:'1'}))<br>RETURN m.odcdnm AS medication,<br>count(*) AS freq<br>ORDER BY freq DESC LIMIT 5	1. Statin<br>2. Nitrate<br>3. Sulfonylurea<br>4. Calcium channel blocker,<br>non-dihydropyridine<br>5. Warfarin	76
8	Gender distribution	MATCH(y:Physical)<br>WHERE y.gender='1'<br>RETURN COUNT(*)<br>MATCH(y:Physical)<br>WHERE y.gender='2'<br>RETURN COUNT(*)	Male : 33030<br>Female : 20811	9<br>4

Supplementary Fig. 6. A screenshot illustrating the Neo4J query result, a bipartite graph of three filtered patients who were treated with Statin medication. The color codes for each node are as follows: Orange-medication, blue-event, green-visit, red-smoking status, yellow-diagnosis, purple-physical, gray-surgery, brown-laboratory, and pink-echo. The edges are pointing outwards from the patient to various node types with different relationship labels on each edge.

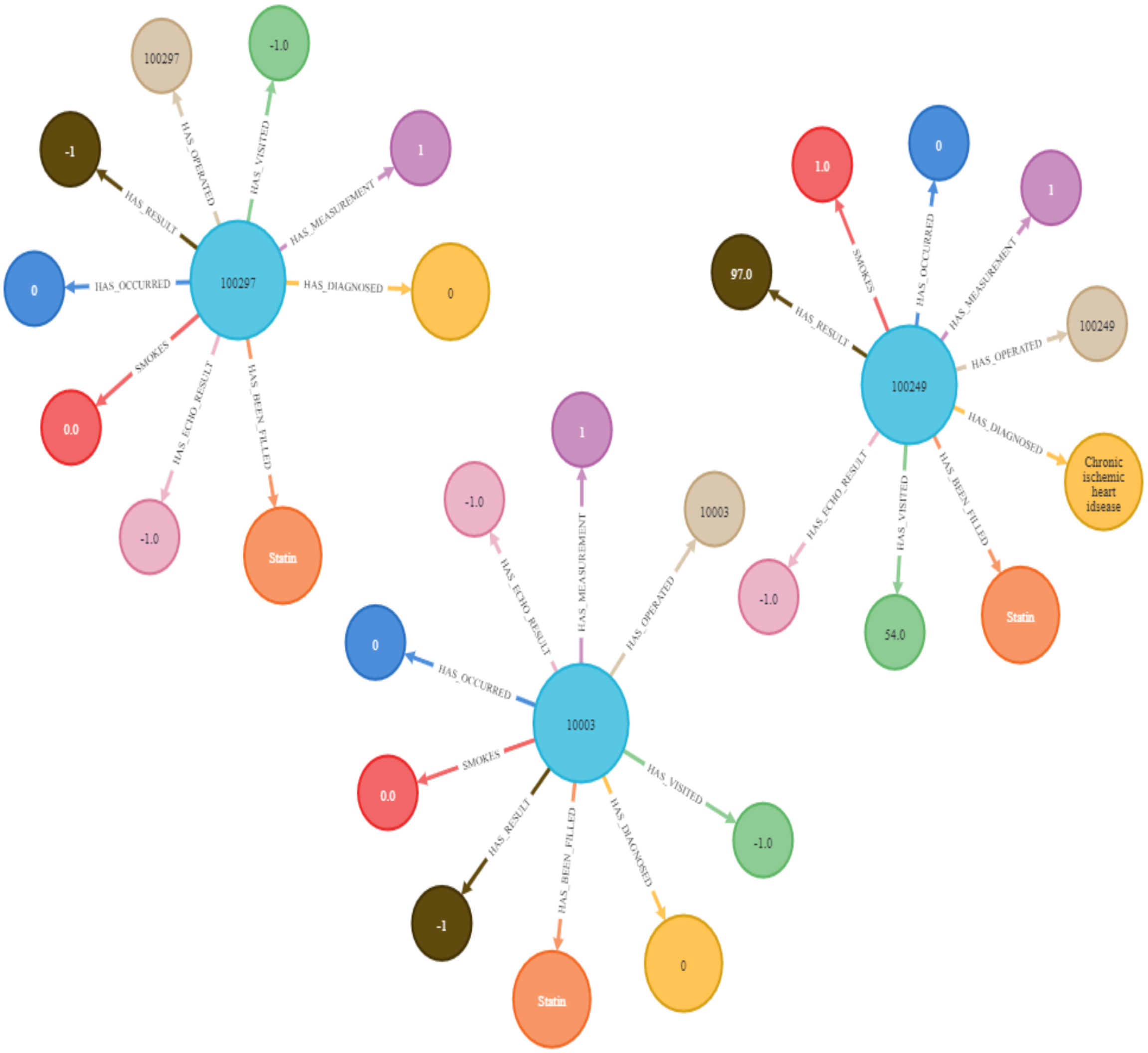

Supplementary Fig. 7. A Neo4j query result capturing the patients associated with any diagnosis who exhibited an event outcome of 1. The blue nodes display the five filtered patients' IDs which are connected to the navy-colored event node representing the event occurrence. In this model, no weight was considered for nodes and edges, yet shows the node size differences according to the various node types.

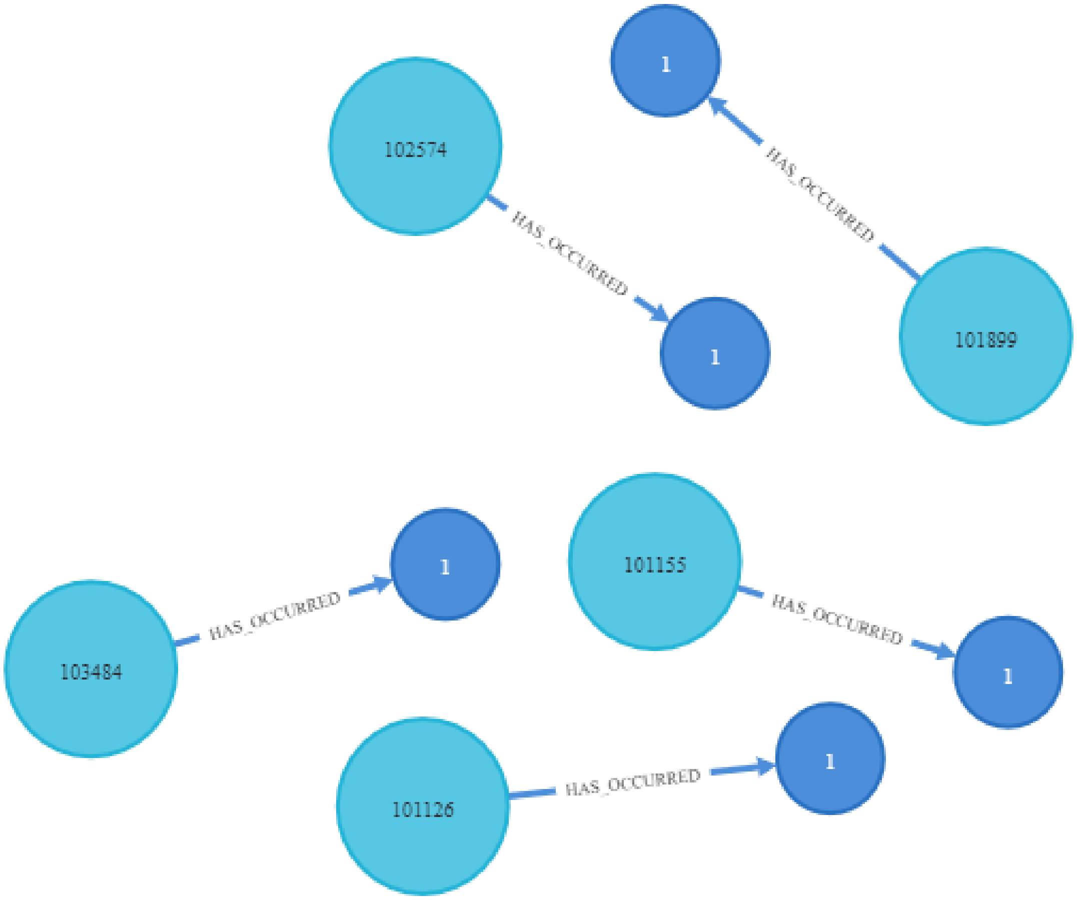

Supplementary Fig. 8. The performance of the selected model’s training history. (a) Accuracy plot; the validation dataset shows a high fluctuation (pink line), while the growth for the training data set is depicted by the blue line until epoch five and shows minimal growth until the end. (b) Loss plot; the loss score for the validation sets is much higher than the loss of the training sets. (C) Distribution plot; the x-axis shows the true binary prediction result exactly on 0 and 1, in contrast to the predicted values which lie between 0 and 1.

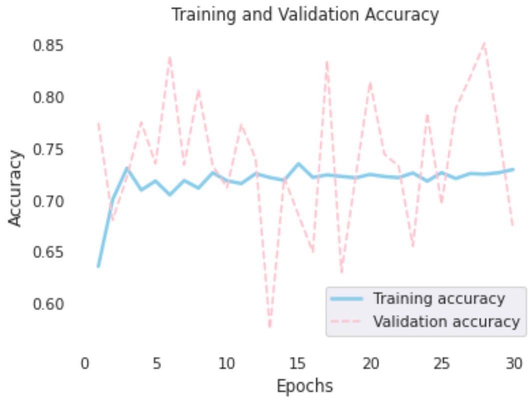

(a)

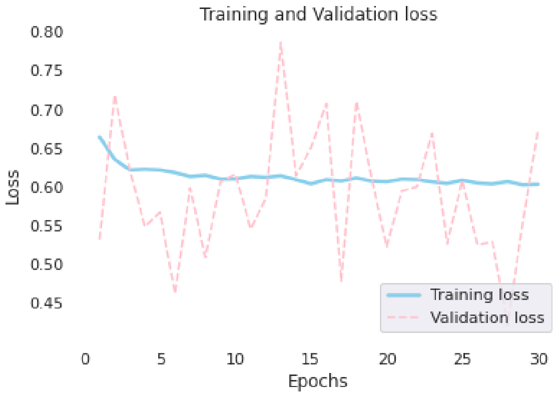

(b)

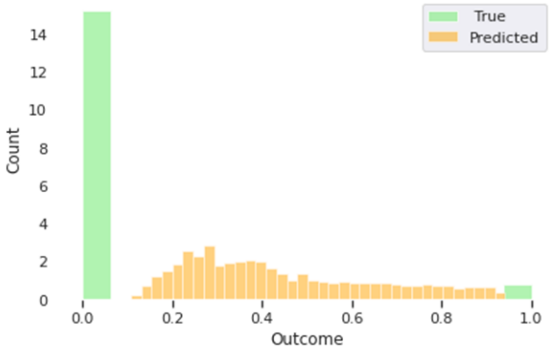

(c)
